# Decoding of Baby Calls: Can Adult Humans Identify the Eliciting Situation from Emotional Vocalizations of Preverbal Infants?

**DOI:** 10.1371/journal.pone.0124317

**Published:** 2015-04-20

**Authors:** Jitka Lindová, Marek Špinka, Lenka Nováková

**Affiliations:** 1 Faculty of Humanities, Charles University, Prague, Czech Republic; 2 National Institute of Mental Health, Klecany, Czech Republic; 3 Department of Ethology, Institute of Animal Science, Prague, Czech Republic; Utrecht University, NETHERLANDS

## Abstract

Preverbal infants often vocalize in emotionally loaded situations, yet the communicative potential of these vocalizations is not well understood. The aim of our study was to assess how accurately adult listeners extract information about the eliciting situation from infant preverbal vocalizations. Vocalizations of 19 infants aged 5-10 months were recorded in 3 negative (Pain, Isolation, Demand for Food) and 3 positive (Play, Reunion, After Feeding) situations. The recordings were later rated by 333 adult listeners on the scales of emotional valence and intensity. Subsequently, the listeners assigned the eliciting situations in a forced choice task. Listeners were almost perfectly able to discriminate whether a recording came from a negative or a positive situation. Their discrimination may have been based on perceived valence as they consistently assigned higher valence when listening to positive, and lower valence when listening to negative, recordings. Ability to identify the particular situation within the negative or positive realm was substantially weaker, with only three of the six situations being discriminated above chance. The best discriminated situation, Play, was associated with high perceived intensity. The weak qualitative discrimination of negative situations seemed to be based on graded perception of negative recordings, from the most intense and unpleasant (assigned to Pain) to the least intense and least unpleasant (assigned to Demand for Food). Parenthood and younger age, but not gender of listeners, had weak positive effects on the accuracy of judgments. Our results indicate that adults almost flawlessly distinguish positive and negative infant sounds, but are rather inaccurate regarding identification of the specific needs of the infant and may normally employ other sensory channels to gain this information.

## Introduction

Vocal communication between the infant and the parent has been studied mainly in terms of precursors of speech development in the infant and early verbal communication, e.g. [1–6]. Less attention was paid to communicative function of non-verbal vocalizations. Therefore, it is still largely unknown how much information adult humans can decode from the vocalizations of preverbal infants. Specifically, the question remains whether parents or other caretakers can identify what type of situation the baby experiences just by listening to its vocalizations.

There is a rich variety of non-verbal vocalization in infants. Scheiner et al. [7] identified twelve categories of preverbal vocalizations: cry, short cry, coo/wail, moan, whoop/squeal, babbling, laugh, hic, groan, croak, raspberry, and ingressive vocalization. Cry is the most studied vocalization, with several cry types being distinguished in the literature, including birth cry, pain cry, hunger cry, attention/separation cry, pleasure cry, mad/angry cry, and startle/auditory cry [8–12]. However, contradictory results have been obtained in studies investigating the ability of listeners to distinguish between different cry types. Whereas Wasz-Hockert et al. [10] and Gustafson and Harris [13] found that adults were able to discriminate between pain and hunger cries, Müller et al. [11] found no discrimination between pain, hunger and startle cries by mothers, and Wiesenfeld et al. [12] found that mothers, but not fathers, were able to discriminate between pain and anger cries.

Few studies have assessed whether adults are able to recognize different situations being experienced by babies based on their non-cry vocalizations. Ricks [14] instructed parents to record their infants in specifically defined situations intended to elicit a request noise, a frustrated noise, a greeting noise and a pleasantly surprised noise. Parents were later able to almost perfectly recognize the different situations when listening to recordings of children other than their own. One methodological problem, beside a low sample size, is that the collection of recordings of different “noise” types was influenced by subjective perceptions of the parents about how such a noise should sound. The findings may, therefore, reflect a shared stereotype among parents about meanings of particular vocalizations rather than accurate estimation of the infants’ emotional states or needs. Papoušek [15] found that situations can be distinguished by adults from voiced sounds of 2-month old infants produced during states of comfort, joyful excitement, and discomfort. However, this study also had methodological issues as 50% of the more ambiguous stimuli were removed during pre-selection and were not used for testing.

In parallel with the investigations on human babies, research is accumulating on young-to-adult vocal transmission of information in nonhuman animals, including information about context and emotional state of the sender [16–19]. A universal system of encoding emotional information into vocalization was proposed for mammals [20]. It is likely that human infants and their caretakers rely on this common mammalian system of communication in the first months of life of the infant when speech is not yet available [21].

Studies on both human and non-human infant vocalizations often investigate how the emotional dimensions of valence and arousal/intensity [22] are encoded in the vocalizations [7,23,24]. Probably on the basis of simple acoustic cues, humans can perceive intensity and valence of vocalization both from infant cry [7,25–28] and animal sounds [29,30,18,19,31]. It is possible that identification of the eliciting context is mediated by the perception of valence and/or intensity but this link has never been properly investigated.

The aim of our study was to establish:

- How accurately adult humans are able to recognise the eliciting situation from recorded infant vocalisations;
- How the accuracy of situation recognition is related to the perceived emotional content of the vocalisations;
- How this ability is affected by gender, parental status, age and education.

## Materials and Methods

### Recordings

We collected recordings of infants aged 5–10 months. According to Stark [32], this age period corresponds to the second half of the third, to the fourth, stage in vocal development of infants. In the third stage, vocal play appears and infants significantly enrich their preverbal vocal repertoire. On the other hand, babbling is not yet a prominent means of communication since it only starts to appear in the fourth stage. Nineteen infants were recorded in 6 situations. The 3 negative situations, Pain (prick at vaccination), Isolation from caregiver and Demand for Food, were chosen because they were expected to elicit the urge in the infants to communicate his/her negative emotional state. Because each of the situations is associated with a specific need (to eliminate the source of pain, reunite with the caregiver, be fed), it could be advantageous for the infants to signal the specific need vocally and for the caregiver to recognize it. In addition, 3 positive situations were chosen, where infant vocalizations may also serve different functions: they either promote vivid interaction with the caregiver (Play), express relief and/or deepen the bond with the caregiver (Reunion with caregiver) or indicate satisfaction after having been fed (After Feeding).

**Fig 1:**
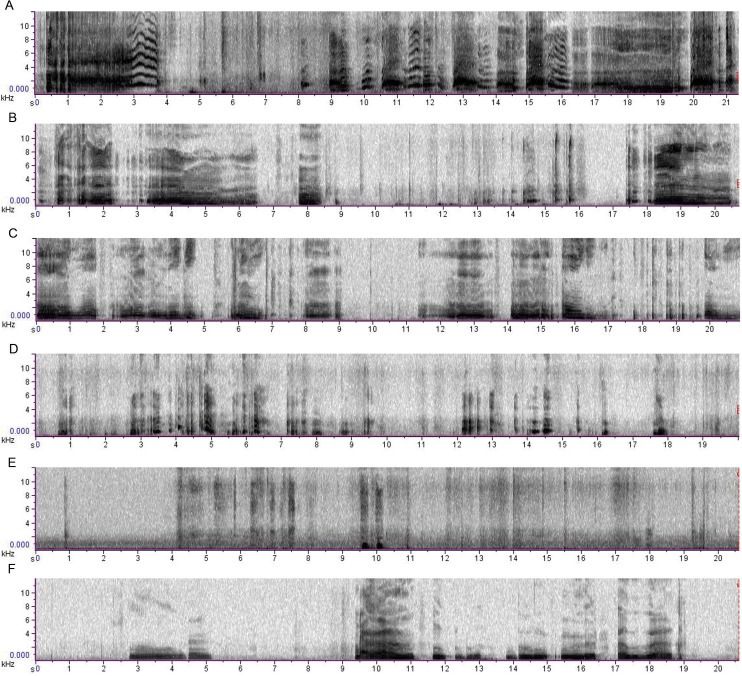
Recordings from the six eliciting situations recognized by the greatest proportion of participants. **Note:** A—Pain, B—Isolation, C—Demand for Food, D—Play, E—Reunion, F—After Feeding.

Mothers (or other caretakers), recruited by snowball sampling, themselves recorded their infants on a Yamaha Pocketrak C24 recorder lent by the researchers for a financial compensation of 500 CZK (apprx. $25). They were instructed orally and in a written form to record at least 30 s of each situation within their everyday routines. Not all caregivers were able to obtain all records, which resulted in a final set of 13 records for Pain, 14 records for Reunion, 17 records for After Feeding and Play, and 18 records for Demand for Food and Isolation (for examples of recordings, see Fig 1). None of the recordings obtained included babbling.

Mothers were verbally informed and instructed, and were given written copies of these information and instructions to take home with them. These instructions guided mothers to obtain their recordings in situations based on daily routines and obligatory procedures in the Czech healthcare (vaccination) only. Mothers were informed about the possibility not to record any situation and not to provide any recordings to researchers, and about anonymous handling of these recordings by researchers. Because it was technically impossible for the experimenters to obtain any recordings or other data about infants that the mothers would not voluntarily collect themselves and actively provide to experimenters, no extra written consent was required. Providing recordings of their babies was accepted as implied consent. Original recordings were stored in a computer under the first name of the infant (and a number in case there were more infants of the same name). Only anonymous non-verbal vocal sequences were used for rating. The research was designed and performed in concordance with legislation in the Czech Republic and was approved (including the implied consent procedure) by the Ethic Committee of the Faculty of Science, Charles University, Prague (No. 2014/17).

To create infant vocal stimuli for rating by adults, 20 s of recorded sound was selected from each infant and situation. The first criterion for the selection of the 20 s interval was noise minimization. When a longer record without noise was present, we chose the first 20 seconds. The recording was cut after the last sound, not in the middle of it, and therefore some recordings used as stimuli were somewhat longer than 20 s. Recordings were played back at their original loudness.

### Listeners

Listeners were recruited through internet advertisements and by snowball sampling. We collected responses from 333 participants who rated at least some records. On average, 55.1 listeners (range, 42–69) rated each recording. One hundred and seventy nine participants completed the rating of intensity and valence in 30 randomly chosen recordings and assigned the eliciting situation in another 30 randomly chosen recordings. Among these 179 listeners, there were 135 females and 44 males. One hundred and six listeners indicated that they had one or more children, and 73 were childless. Age range was 16–68 (median 29).

### Ratings

The rating form and questionnaire was created in Qualtrics (http://www.qualtrics.com/). First, participants indicated their sex, age, educational level and income category, parental status, professional experience with babies and personal experience with babies other than their own. Secondly, they rated 30 randomly selected recordings for intensity and valence on 7-point Likert scales presented simultaneously with each record. The instruction stated:*”Please judge the intensity and pleasantness of given infant‘s vocal display on the presented scales”*. Participants rated intensity on an increasing scale from 1 to 7 and valence on a scale from 1 (very unpleasant) to 7 (very pleasant). Thirdly, they assigned another 30 randomly selected recordings to the eliciting situation. The situations were described as: *Pain—reaction of the child to a painful procedure*, *such as a prick with a needle during vaccination*. *Play/pleasant interaction with mother—any activity with mother (or another close person)*, *which the infant enjoys a lot*. *Isolation from mother—the infant stays alone*, *or with someone it does not know or does not like much*, *and shows displeasure*. *Reunion with mother—after a period when the infant was calm*, *but isolated from mother*, *it expresses joy from reunion with mother*. *Demand for food/restriction of food—the infant knows that breastfeeding is approaching and it is nervous that feeding has not started yet (mostly because the mother is not yet ready)*. *After feeding or during feeding—reaction of the infant to having been fed and being satisfied*. Participants were required to choose one of these six situations for each of 30 randomly selected records.

### Analyses

To assess listeners’ gross discrimination of whether the situation was negative or positive, we counted how many times the stimuli recorded in any of the three negative situations were assigned to any negative situation, and analogously for positive situations. To investigate discrimination among specific eliciting situations, the stimulus (i.e. specific combination of infant and situation) was set as the unit of analysis. Using Wilcoxon signed rank tests performed separately for each recording situation, we compared the proportions of listeners (out of all listeners who made correct negative/positive gross discrimination) that assigned the stimulus to the correct situation against the 1/3 proportion expected by chance. Next, we tested, at the stimulus level and for each of the six situations separately, the relationship between the proportion of correct assignments to the situation and the perceived intensity and valence of the stimulus, using Kendall’s Tau correlation. To investigate which participants' characteristics influenced their ability to recognize the situations, the participant was set as the unit of analysis. We ran separate general linear models for overall recognition, recognition of the positive and negative situations, and for each situation individually, with the dependent factor being proportion of correct responses and independent predictors being sex, age, having a child/children and education. We did not include professional and personal experience with children, which showed no predictive power in any model, or income, which was highly correlated with age.

## Results

### Discrimination among eliciting situations

Listeners were strikingly accurate at recognizing whether the playback came from a negative or a positive situation (Table 1, last two columns). Vocalizations recorded in one of the negative situations were almost always (> 98% of cases), assigned the correct (negative) valence, i.e. assigned to a negative situation. Among the vocalizations recorded in positive situations, the “false negative” assignments were somewhat more frequent, yet even for them, the correct gross judgement was made in 84–90% of cases.

**Table 1:** Proportions of listeners assigning playbacks (recorded in six situations—rows) to the six situations (first 6 columns) and to any positive or negative situation (last two columns).

Assigned:	Pain	Isolation	Demand for Food	Play	Reunion	After Feeding	A negative situation	A positive situation
Recorded Pain	.42* (.12/.71)	.28** (.18/.51)	.17 (.07/.26)	.00 (.00/.01)	.00 (.00/.02)	.00 (.00/.03)	1*** (.96/1)	.00*** (.00/.04)
Recorded Isolation	.23 (.07/.36)	.41*** (.30/.51)	.29** (.20/.52)	.00 (.00/.02)	.00 (.00/.00)	.00 (.00/.01)	1*** (.96/1)	.00*** (.00/.04)
Recorded Demand for Food	.13 (.07/.20)	.40*** (.32/.49)	.35*** (.28/.49)	.00 (.00/.00)	.01 (.00/.04)	.00 (.00/.02)	.98*** (.93/1)	.02*** (.00/.07)
Recorded Play	.00 (.00/.02)	.02 (.01/.06)	.09 (.03/.16)	.51*** (.32/.78)	.14 (.08/.24)	.07 (.02/.30)	.14*** (.05/.28)	.86*** (.72/.95)
Recorded Reunion	.00 (.00/.00)	.03 (.02/.18)	.10 (.07/.19)	.18 (.08/.36)	.18 (.11/.35)	.31* (.10/.59)	.16*** (.08/.32)	.84*** (.68/.92)
Recorded After Food	.00 (.00/.00)	.04 (.01/.08)	.05 (.03/.14)	.20 (.11/.29)	.20 (.08/.34)	.36** (.20/.67)	.10*** (.06/.26)	.90*** (.75/.94)

Median and 25/75 percentiles across playbacks are given in each cell. Medians significantly above random assignment are denoted as *p<.05, ** p<.01, ***p<.001 (Wilcoxon non-parametric T-test). The random assignment was set to 1/6 for the individual 6 situations and to 1/2 for the positive/negative distinction.

Five of the six situations were recognized above the randomly expected success of 1/6 (Table 1, first six columns). However, this could have been mainly due to the more general negative-positive discrimination rather than to recognition of the specific situations. Therefore, we next examined whether people discriminated among the specific negative and positive situations.

Table 2 shows that listeners were also performing some discrimination among the three negative and among the three positive situations, although here the accuracies were relatively weak. Three situations (Play, Isolation, After Feeding) were recognized above the 1/3 chance level. Play was the situation that was recognized with the highest accuracy and on most occasions. In Isolation and After Feeding, the recognition was weaker as less than half of the recordings were correctly assigned. The other three situations were either not recognized significantly (Pain, Reunion) or mistaken for another situation (Demand for Food mistaken for Isolation).

**Table 2:** Proportions of listeners assigning playbacks (recorded in three negative and three positive situations—rows) to the situations (columns).

		Assigned negative			Assigned positive		
		Pain	Isolation	Demand for Food	Play	Reunion	After Feeding
Recorded negative	Pain	**.**42 (.13/.71)	.30 (.18/.58)	.17 (.08/.32)			
	Isolation	.13 (.07/.36)	**.43** ^*^ (.31/.52)	.29 (.20/.55)			
	Demand for Food	.13 (.08/.22)	**.43** ^**^ (.33/.52)	.38 (.28/.56)			
Recorded positive	Play				**.65** ^**^ (.37/.85)	.21 (.11/.26)	.08 (.02/.36)
	Reunion				.20 (.12/.42)	.32 (.14/.43)	.39 (.24/.67)
	After Feeding				.27 (.12/.37)	.22 (.10/.41)	**.45** ^*^ (.26/.78)

Only recordings that have been correctly assigned to negative or positive domain are used in this table. Median and 25/75 percentiles across playbacks are given in each cell. Medians significantly above chance proportion of 1/3 are denoted as *p<.05, ** p<.01, ***p<.001 (Wilcoxon non-parametric T-test).

### Perceived valence and intensity and its relation to discrimination success

**Fig 2:**
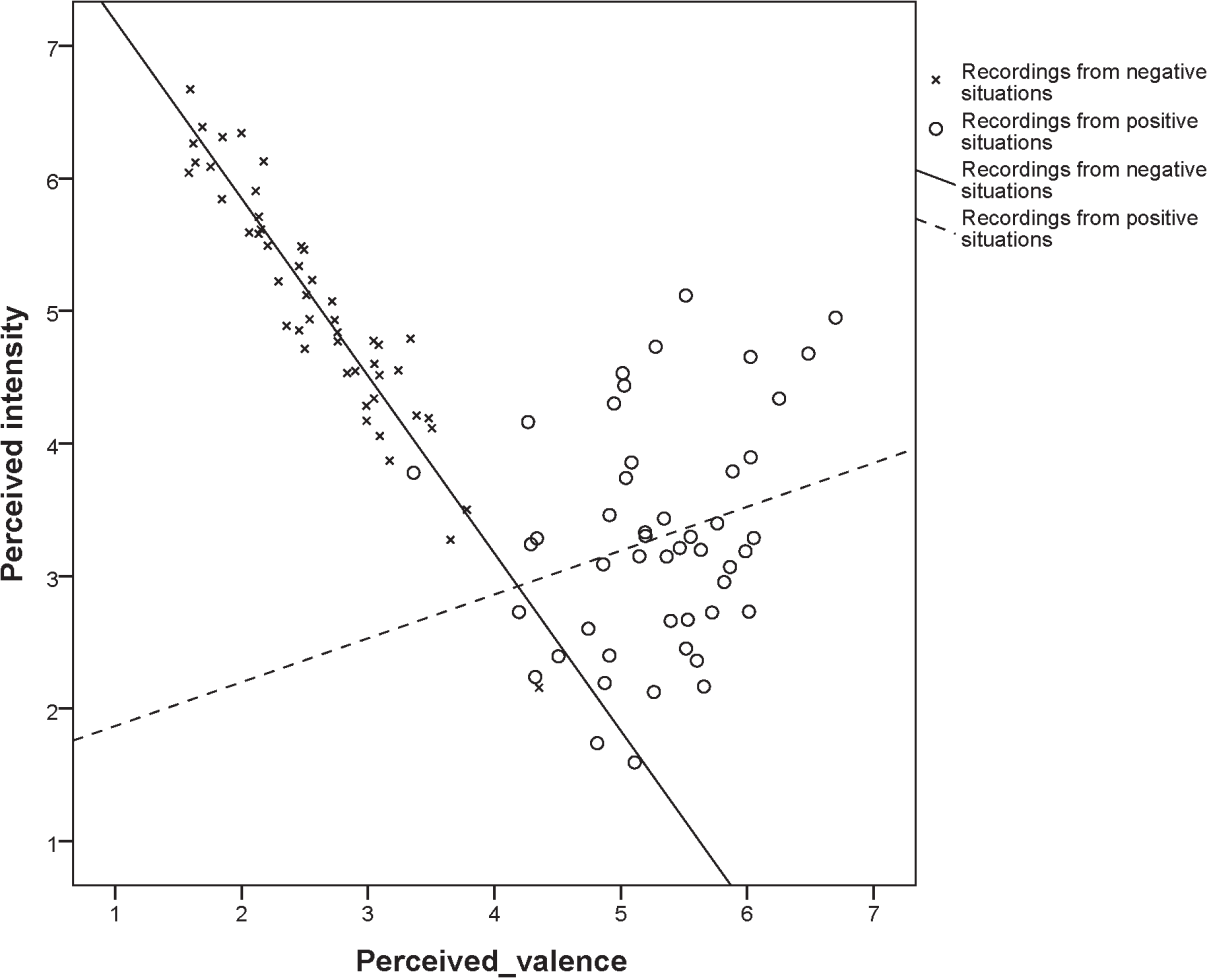
Perceived valence and intensity in infant recordings from positive and negative situations. **Note:** Data points represent individual recordings and show average intensity and valence judgments by participants. Regression lines for recordings obtained in positive and negative situations shown.

There was a large variation in both the perceived valence and the perceived intensity among the individual recordings (Fig 2). Among the recordings made in the three negative situations, perceived valence and intensity correlated strongly negatively with each other (Kendall Tau = -.80, p < 0.001). In contrast, there was no correlation between intensity and valence among the recordings from positive situations (Tau =. 16, p =. 11).

**Fig 3:**
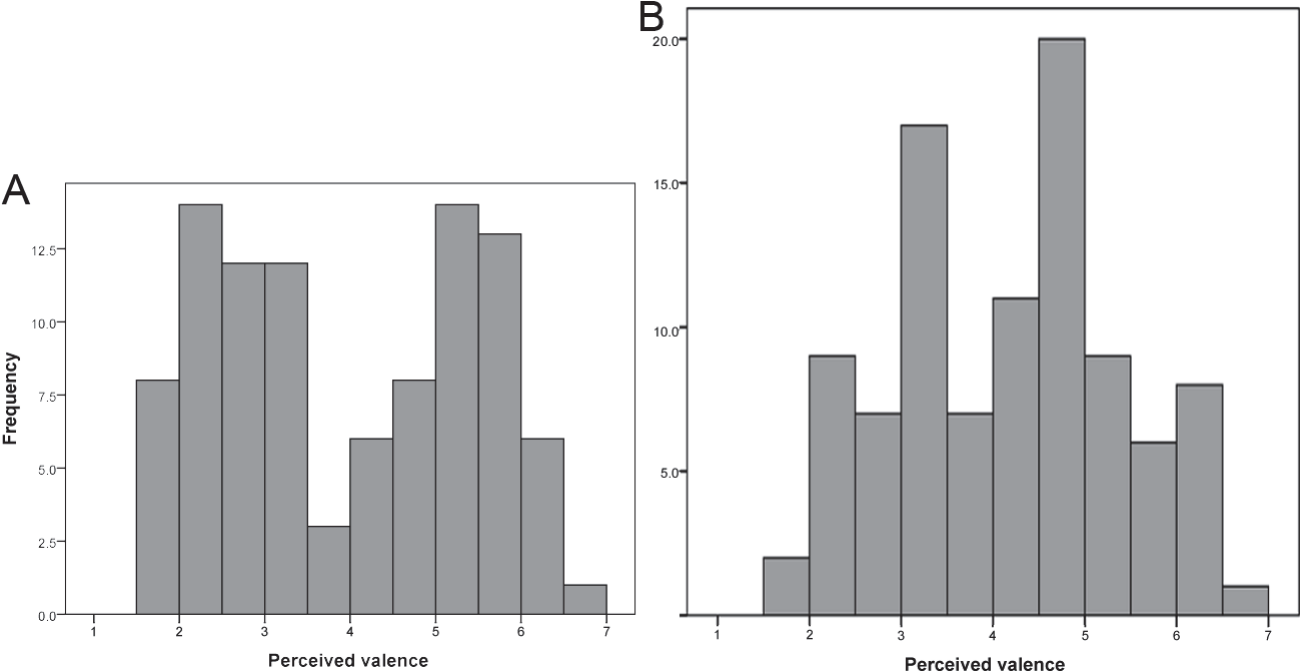
Bimodal distribution of the emotional valence judgments of recordings (A) and unimodal distribution of the intensity judgments of recordings (B).

Conspicuously, the perceived valence of vocalizations from negative situations (range 1.59–4.43) and from positive situations (range 3.36–6.70) did not overlap at all, with the exception of one single pain recording and one single reunion recording (Fig 2). Moreover, the distribution of the valence values was clearly bimodal, with relatively few vocalizations judged as neutral (Fig 3A). In contrast, the perceived intensity of vocalizations from positive and negative situations overlapped widely and the distribution of intensity values was unimodal (Fig 3B).

**Fig 4:**
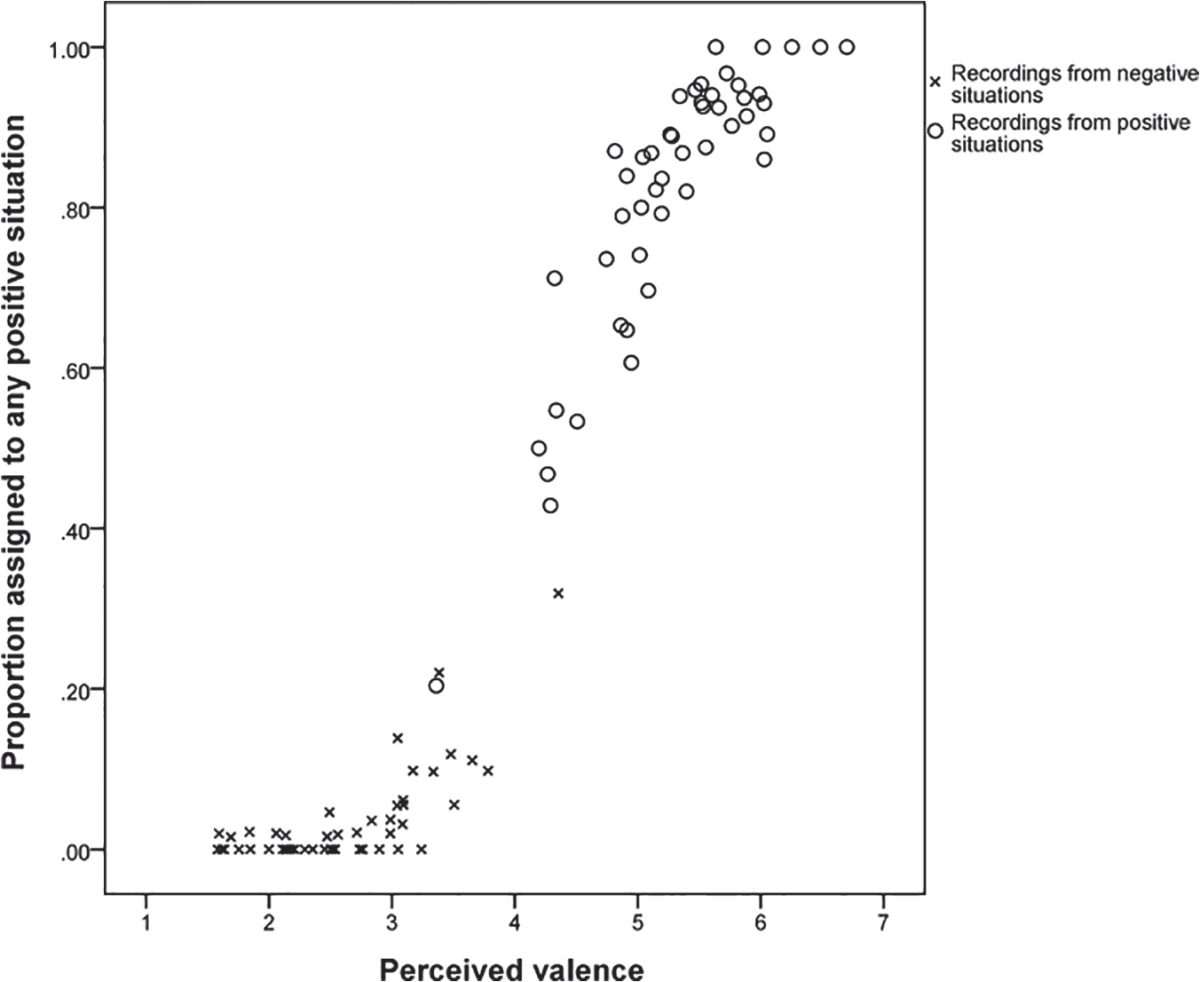
Association between emotional valence judgments and assignment of recordings to a positive eliciting situation by listeners.

At the level of recordings, there was a clear relationship between the perceived valence and the gross discrimination between negative and positive situations, i.e. assignment of a recording to one of the three negative situations or to one of the three positive situations. Recordings with negative perceived valence (< 4.0) were almost always assigned to either Pain, Isolation or Demand for Food situation while recordings of valence > 5 were prevailingly assigned to Play, Reunion, or After Feeding situation. Only the few recordings with valence between 4 and 5 got mixed assignment between negative and positive situations (Fig 4).

**Fig 5:**
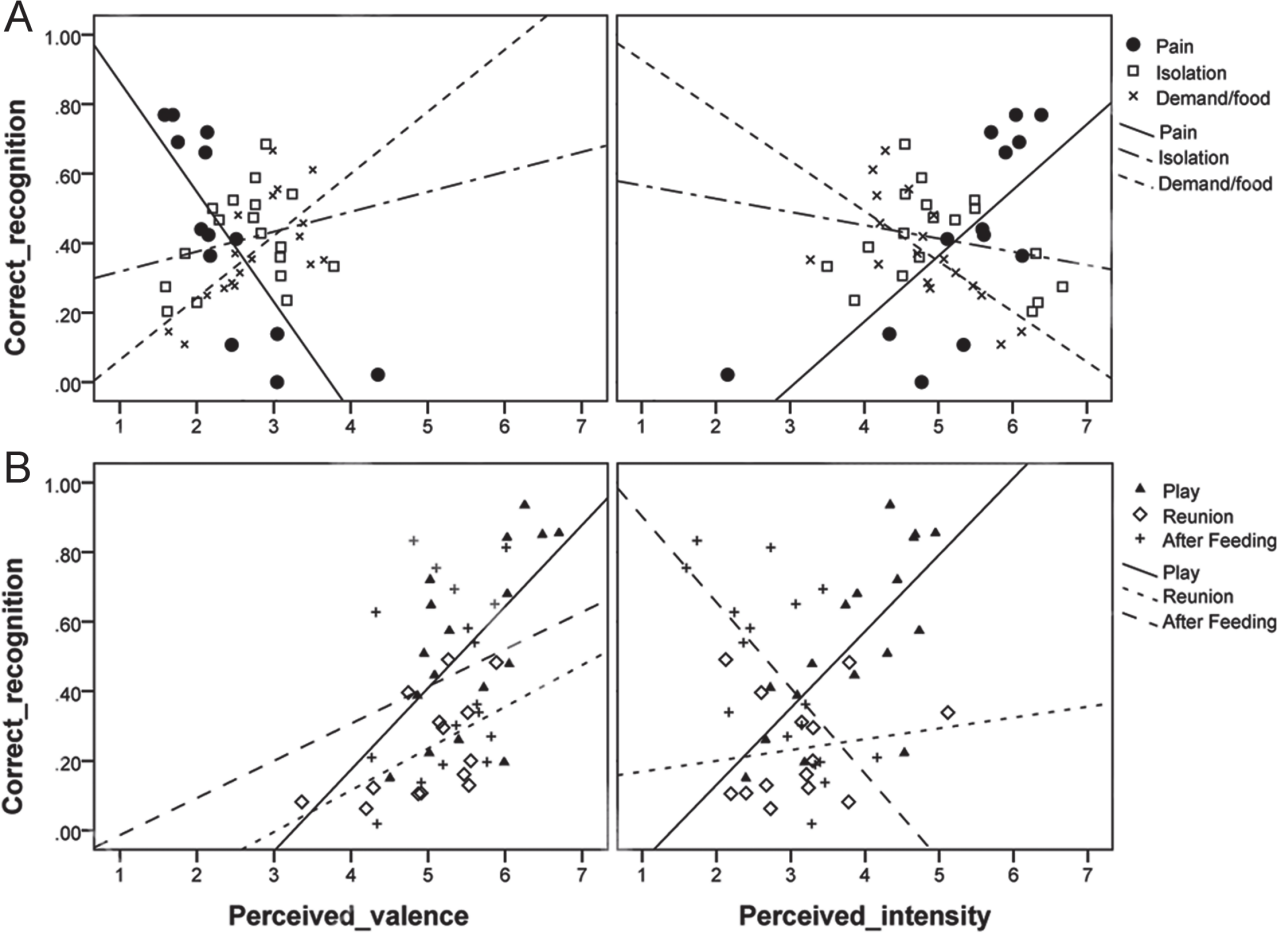
Association between emotional valence and intensity judgments, respectively, and correct recognition of the particular negative (A) and positive (B) situation by listeners.

Next we tested if the perceived intensity and valence of recordings taken in a specific situation correlated with the probability that the situation would be correctly recognized. As Fig 5A shows, Pain was recognized more often in recordings perceived as less pleasant and more intense (Kendal Tau = -.76 and. 61, respectively, *p*s <. 01). Conversely, Demand for Food was assigned correctly in those recordings that were perceived as more pleasant and less intense (.55 and-.60, respectively, *p*s <. 01). This indicates that participants used pleasantness and intensity as important cues to discriminate among negative situations, especially between Pain and Demand for Food.

The Play situation was recognized more often in recordings perceived as more pleasant and more intense (.44, p <. 05 and. 59, p <. 01, respectively, Fig 5B). Correct assignments of the Reunion situation were given more often when those recordings were perceived as more pleasant (.43, p <. 05). After Feeding was identified more often in recordings perceived as less intense (-.44, p <. 05). Thus, intensity seemed to be a cue that facilitated discrimination among the positive situations along the gradient Play—Reunion—After Feeding, while perceived valence also played a role.

### Participants’ characteristics and discrimination success

Furthermore, we were interested if any participants’ characteristics affected the accuracy of recognition of infant vocalizations. Overall, these characteristics explained only small proportions of inter-individual variability in accuracy of situation recognition (η between. 017 and. 055). Having own children improved the overall recognition (β =. 076, F_1,176_ = 7.84, p =. 006, η =. 044, mean of. 42 correct assignments by listeners with children,. 39 by childless listeners), the recognition of the three positive playback situations (β =. 092, F_1,176_ = 5.46, p =. 021, η =. 031,. 43 correct assignments by listeners with children,. 39 by childless listeners), the identification of the Play situation (β =. 16, F_1,179_ = 5.26, p =. 023, η =. 029,. 58 correct assignments by listeners with children,. 54 by childless listeners), and identification of the Demand for Food situation (β =. 13, F_1,180_ = 4.28, p =. 040, η =. 024,. 41 correct assignments by listeners with children,. 34 by childless listeners). Younger listeners were better than older ones at overall recognition (β = -.002, F_1,176_ = 4.79, p =. 030, η =. 027) and in identification of the Play situation (β = -.005, F_1,179_ = 4.85, p =. 029, η =. 027). There were no significant effects of sex on recognition accuracy. For all other effects of participant characteristics on discrimination success, p >. 1 and η <. 02. The complete results are presented in S1 Table.

## Discussion

Participants were highly efficient at discriminating between positive and negative situations. In particular, vocal signals from infants in negative situations were almost never falsely discounted as positive cues. On the other hand, vocal signals from positive situations were sometimes (in 15–20% of cases) mistaken for cues of a negative situation. Similarly Papoušek [15] found higher recognition of negative over positive situations. This bias in adult human listeners may be adaptive as the cost of neglecting a signal from an infant in distress is higher than the cost of false alarm in the case of a positive signal.

The accurate discrimination between negative and positive situations may be based on the perceived quantitative valence (unpleasantness/pleasantness) of the playback. There was almost no overlap in the perceived pleasantness of the playbacks coming from the negative and positive situations, with a division point of 4.00 at the neutral midpoint of the scale from very unpleasant to very pleasant. Thus the listeners were able to apprehend all vocalizations coming from the three negative situations as unpleasant and all the calls from the positive situations as pleasant despite the fact that they made the valence judgement through the questionnaire before they knew anything about the situations in which the vocalizations had been recorded. Moreover, valence judgements were concentrated at both ends of the pleasantness/unpleasantness scale with relatively few recordings being given values in the middle of the valence scale. Finally, at the level of individual recordings, there was a close relationship between the estimated valence and assignment to either positive or negative realm, in spite of the fact that the estimation of valence and the judgement of the situations were done by different listeners for any particular recording. These results suggest that adult humans categorize baby vocalizations rather dichotomously as either emotionally positive or emotionally negative. Through this categorization, adult listeners are able to tell apart situations in which the baby is in distress and thus needs care or protection from those situations in which the baby is content, relieved or joyful. It remains to be investigated whether vocalizations in more neutral situations would fill the middle in valence judgement. Nevertheless, the bimodal distribution found in our study may still correspond to the overall distribution of baby vocalizations in reality as infants presumably vocalize mainly in valenced situations rather than in situations where nothing either positive or negative is happening to them.

In contrast to the highly accurate discrimination between negative and positive situations, the identification of the particular situation within the negative or the positive realm was substantially weaker. The identification of the particular negative situation, Pain, Isolation from mother and Demand for Food, was somewhat above the chance level for all three situations, but in all three cases, they were also interchanged with another negative situation. Pain was often misclassified as Isolation, and Isolation with Demand for Food were often interchanged with each other. Pain and Demand for Food were rarely confused, in agreement with the studies of Wolff [33] and Gustafson and Harris [13]. Among the positive situations, Play with mother was recognized correctly by a significant proportion of the judgers, and was not interchanged with any other situation while After Feeding and especially Reunion were often mistaken for another positive situation. The low recognition of Reunion seems to contradict Ricks’ study [14], where he found that infant’s greeting noise was reliably recognized by parents and strangers. In Ricks’ study, greeting sounds were recorded in a slightly different situation, namely on waking of the infant when it saw his/her mother coming to his/her cot. Moreover, the parents knew that they should record a “greeting noise” and could therefore have projected their expectations about this sound when deciding which sounds to record.

The overall weaker recognition of particular situations was due to large variability in correct assignments across recordings. For instance, among the Pain recordings, some had a recognition rate of almost 80%, while others were close to 0%, and the Play recording recognition rates ranged between 15 and 95%. Since these highly varying recognition rates were based on responses of a large number of independent listeners, they are not results of random variation but rather reflect genuine and profound differences among the individual recordings: some were easy for people to identify while others mislead all listeners into wrong situation identification.

Why were some recordings misclassified very rarely while others were mistaken very often? Our data indicate that this was related to the perceived valence and intensity of the particular recording. For the negative situations, the discrimination strategy seemed to be based on the highly inter-correlated intensity and valence perceptions. When people heard a recording that they perceived as intensely unpleasant, they were drawn to assign it to the Pain situation, and were usually right. Conversely, less intense and less unpleasant playbacks were assigned to Demand for Food or Isolation situations. This corresponds to the previous finding that cry vocalizations served as a graded signal of the infant’s level of distress [34]. This discrimination strategy led to mistakes when recordings from the Pain situation were perceived as only mildly unpleasant or when non-Pain recordings were perceived as intensely unpleasant. A possible functional explanation for these tendencies is that those specific playbacks that were judged as intensely negative indeed signalled higher distress of the child independently of the eliciting situation and, therefore, it was adaptive for the adults to identify the situation as painful (i.e. dangerous) for the child and react quickly. Conversely, it might be that some infants did not feel the prick during the vaccination and the listeners correctly noted that the level of distress in the vocalisation was low and classified it into one of the other negative situations.

Among the positive situations, Play recordings perceived as more intense and After Feeding recordings perceived as less intense were recognized by a higher proportion of judgers, suggesting that people might use a similar mechanism for discriminating among the positive situations as in the negative situations. Here, however, perceived valence and intensity were uncorrelated and perceived intensity seemed to play a stronger role in the assignment. It is possible that, after identifying that the vocalization belonged to the positive realm, the information that the adults extracted concerned how much arousal the child was experiencing at the moment.

The results indicate that the ability of adult humans to discriminate between situations eliciting infant vocalisations consists of two processes. First, the gross distinction whether the situation is generally negative or generally positive for the baby is made with great accuracy. Second, estimation of the specific situation is performed, possibly based on the perceived intensity and valence of the sound, but this discrimination is uncertain and rather unreliable. Finer distinction about the cause of the infant calling is probably made after closer inspection and combination of visual, acoustic and other cues. This interpretation is fully in line with the summary of older studies by Murray [34] and with the findings of Gustafson and Harris [13] that the first reaction of caretakers to a cry of a baby is to pick it up, independent of the actual cause of the crying. This conclusion also agrees with previous studies of human assessment of crying vocalisations. Müller et al. [11] interpreted that the cry itself carries little information about the eliciting situation, but serves only to alert the mother who then infers the cause of the cry from other cues. Similarly, Gustafson and Harris [13] stated that “cry sounds communicate information about the general distress level of the infant better than information about specific causes” (p. 144) and Zeifman [8] concluded her overview of published research by stating that “adult listeners easily distinguish between crying of varying intensity level, but are unable to discriminate between different cries elicited under different circumstances” (p. 277).

Among the properties of the individual listeners, being a parent enhanced the ability to recognize situations from infant vocalizations. In particular, the Play situation, the Demand for Food situation as well as the positive situations in general were better identified by parents than by non-parents. Previous studies showed the positive role of parity in guessing why an infant was crying [35]. Our study which was not limited to cry recordings, shows that parity also improves recognition of non-cry negative and positive sounds, as already indicated in a small sample by Papoušek [15]. Perhaps in the case of parents, discrimination might not rely so heavily on the perceived general valence and may become more specific with experience. Younger age also contributed positively to situation recognition abilities, albeit less so than parenthood as it only influenced the overall recognition and the Play situation identification. Even with our quite extensive data set, we could not detect a significant effect of listeners’ sex on discrimination ability, in agreement with Papoušek [15]. This indicates that, if a sex effect exists, it must be quantitatively small. Leger et al. [28], too, found no gender effect on the perception of emotions in infant cries and Wiesenfeld et al. [12] and mainly Gustafsson et al. [36], who controlled for experience of both sexes with parenting, found no difference in the ability of mothers and fathers to recognize cries of their own babies from cries of other babies. Wiesenfeld et al. [12] also found that mothers, but not fathers, were able to discriminate between two types of cries, possibly thanks to a greater experience of mothers with caring for infants compared to fathers. Our results thus support the hypothesis that men and women are equally well predisposed to perceive and decode information from baby vocalisations. According to a recent study on marmosets, this might also hold for other mammals with biparental care [37]. Education level had no effect, with the exception of the peculiar negative influence of identification of Reunion recordings. We have no explanation for this particular result, other than that it might have arisen purely by chance. Most importantly, the combined explanatory power of parenthood, age, sex and education did not surpass 5% in any of the models. Hence, the inter-individual variability in decoding skills of infant vocalisation remains largely unexplained by the systemic factors examined in this study. This is in agreement with the study of Leger et al. [28] who found that parental experience explains much less variability in the perceived emotion than other factors such as the age of the child.

In a recent similarly designed study [31,18], human listeners were asked to decode infant vocalizations of another mammalian species, namely the domestic pig. There are close similarities in the results of both studies. As in the current study, perceived valence was strongly negatively correlated with perceived intensity for negative situations, and there was no relationship between the two emotional dimensions for positive situations. In the piglet-playbacks study, too, the recognition of calls from the Pain situation correlated positively with perceived intensity while the recognition of the After Nursing situation decreased with perceived intensity. In another parallel between the two studies, individual properties of listeners such as gender, personality and attitude towards animals did not significantly affect either the recognition of situations. These similarities indicate that the perceptual and cognitive mechanisms that evolved for decoding of baby vocalizations may be also employed by adult humans for decoding of infant animal sounds. If so, this would resonate with theories of shared emotional systems across mammalian species, first postulated by Darwin in his theory of common emotional expressions across mammals [38] and later described for emotional vocalizations in Morton’s Motivation-Structural Rules [20,24]. Empirical studies indicate that cross-species similarities in encoding and decoding of adult emotional vocalizations exist, but are rather loose [39,40]. In contrast, similarities in mammalian infant vocalizations are strong enough to elicit a response by distant species [41]. This is possibly due to less voluntary control and higher overlap between the evolutionary interests of the infant and the parent than it is the case in communications between adult individuals. For the case of humans, it was shown, indeed, that individuals respond more strongly to infant cries than to cries of adults [42].

In summary, adult humans were able to decode perfectly whether an infant vocalisation came from a positive or negative situation. The listeners also assessed sensitively the degree of urgency in the negative situations and the degree of intensity in the positive situations. In contrast, the discrimination of the specific eliciting situations was relatively inaccurate indicating that finer distinction of the actual cause is probably made after closer inspection. Furthermore, we found that parenthood and, to a lesser extent, younger age made the discrimination somewhat more accurate, but these effects were weak. The lack of significant gender effect on the discrimination abilities in our extensive study indicates that an advantage of women over men in interpretation of infant vocalisations, although it might exist, is quantitatively small. The study substantially expands previous knowledge based on analyses of cry vocalisations alone. Further research is in progress by our group to determine the role of acoustic parameters of the particular calls on human judgements about the situation, intensity and valence, extending previous bioacoustics analyses that were limited to infant cry vocalizations [27,26].

## Supporting Information

S1 Table Effects of raters’ characteristics of their recognition accuracy. (DOCX)
