# Supplementary material for: Decoding of Baby Calls: Can Adult Humans Identify the Eliciting Situation from Emotional Vocalizations of Preverbal Infants?
Source: PLoS One. 2015 Apr 20;10(4):e0124317. doi: 10.1371/journal.pone.0124317 (PMC4403804; doi:10.1371/journal.pone.0124317)
Supplement: S1 Table — (DOCX) [file pone.0124317.s001.docx]

**Table S1. Effects of raters’ characteristics of their recognition accuracy**

| **Overall recognition** | b | F | df | p | η | mean 1 | mean 2 |
| --- | --- | --- | --- | --- | --- | --- | --- |
| Sex | .022 | .24 | 1,176 | .63 | .001 | female=.41 | male=.39 |
| Children yes/no | .076 | 7.84 | 1,176 | .006** | .044 | parent=.42 | non-par.=.39 |
| Age | -.002 | 4.79 | 1,176 | .030* | .027 |  |  |
| Education | .000 | .00 | 1,176 | .97 | <.001 |  |  |
| Sex x Children | -.027 | .63 | 1,176 | .43 | .004 |  |  |
| **Recognition of pos. situations** |  |  |  |  |  |  |  |
| Sex | .012 | .02 | 1,176 | .89 | <.001 | female=.41 | male=.40 |
| Children yes/no | .092 | 5.46 | 1,176 | .021* | .031 | parent=.43 | non-par.=.39 |
| Age | -.002 | 1.94 | 1,176 | .17 | .011 |  |  |
| Education | -.004 | .15 | 1,176 | .70 | .001 |  |  |
| Sex x Children | -.031 | .41 | 1,176 | .52 | .002 |  |  |
| **Recognition of neg. situations** |  |  |  |  |  |  |  |
| Sex | .030 | .64 | 1,176 | .42 | .004 | female=.41 | male=.38 |
| Children yes/no | .058 | 2.31 | 1,176 | .13 | .013 | parent=.41 | non-par.=.40 |
| Age | -.002 | 2.91 | 1,176 | .090 | .017 |  |  |
| Education | .003 | .11 | 1,176 | .74 | .001 |  |  |
| Sex x Children | -.021 | .20 | 1,176 | .65 | .001 |  |  |
| **Recognition of Play** |  |  |  |  |  |  |  |
| Sex | -.019 | 1.79 | 1,179 | .18 | .010 | female=.55 | male=.58 |
| Children yes/no | .16 | 5.26 | 1,179 | .023* | .029 | parent=.58 | non-par.=.54 |
| Age | -0.005 | 4.85 | 1,179 | .029* | .027 |  |  |
| Education | .006 | .19 | 1,179 | .66 | .001 |  |  |
| Sex x Children | -.069 | .79 | 1,179 | .38 | .005 |  |  |
| **Recognition of Reunion** |  |  |  |  |  |  |  |
| Sex | .21 | .19 | 1,178 | .66 | .001 | female=.23 | male=.21 |
| Children yes/no | .017 | .07 | 1,178 | .80 | <.001 | parent=.24 | non-par.=.22 |
| Age | .002 | .86 | 1,178 | .36 | .005 |  |  |
| Education | -.04 | 8.07 | 1,178 | .005** | .045 |  |  |
| Sex x Children | -.007 | .01 | 1,178 | .93 | <.001 |  |  |
| **Recognition of After Food** |  |  |  |  |  |  |  |
| Sex | .033 | .25 | 1,179 | .62 | .001 | female=.45 | male=.42 |
| Children yes/no | .11 | 2.92 | 1,179 | .09 | .017 | parent=.48 | non-par.=.42 |
| Age | -.003 | 1.75 | 1,179 | .19 | .010 |  |  |
| Education | .025 | 2.61 | 1,179 | .11 | .015 |  |  |
| Sex x Children | -.023 | .08 | 1,179 | .78 | <.001 |  |  |
| **Recognition of Pain** |  |  |  |  |  |  |  |
| Sex | .076 | 2.22 | 1,177 | .14 | .013 | female=.45 | male=.38 |
| Children yes/no | .037 | .20 | 1,177 | .66 | .001 | parent=.44 | non-par.=.42 |
| Age | .000 | .004 | 1,177 | .95 | <.001 |  |  |
| Education | .001 | .005 | 1,177 | .94 | <.001 |  |  |
| Sex x Children | -.024 | .08 | 1,177 | .78 | <.001 |  |  |
| **Recognition of Isolation** |  |  |  |  |  |  |  |
| Sex | -.004 | .054 | 1,178 | .82 | <.001 | female=.41 | male=.40 |
| Children yes/no | .012 | .018 | 1,178 | .89 | <.001 | parent=.39 | non-par.=.42 |
| Age | -.003 | 1.91 | 1,178 | .17 | .011 |  |  |
| Education | .005 | .12 | 1,178 | .73 | .001 |  |  |
| Sex x Children | -.009 | .015 | 1,178 | .90 | <.001 |  |  |
| **Recognition of Demand f/Food** |  |  |  |  |  |  |  |
| Sex | .024 | .12 | 1,180 | .73 | .001 | female=.38 | male=.35 |
| Children yes/no | .13 | 4.28 | 1,180 | .040* | .024 | parent=.41 | non-par.=.34 |
| Age | -.003 | 1.54 | 1,180 | .22 | .009 |  |  |
| Education | .001 | .003 | 1,180 | .96 | <.001 |  |  |
| Sex x Children | -.019 | .055 | 1,180 | .81 | <.001 |  |  |
